# Upregulation of Toll-like receptor (TLR) expression and release of cytokines from P815 mast cells by GM-CSF

**DOI:** 10.1186/1471-2121-10-37

**Published:** 2009-05-09

**Authors:** Haiwei Yang, Jifu Wei, Huiyun Zhang, Liyan Lin, Wei Zhang, Shaoheng He

**Affiliations:** 1 Clinical Research Center, the First Affiliated Hospital of Nanjing Medical University, Nanjing, Jiangsu 210029, PR China; 2 Allergy and Inflammation Research Institute, Shantou University Medical College, Shantou, PR China

## Abstract

**Backgroud:**

Recently, mast cells have been recognized to express several Toll-like receptors (TLRs) on their membrane surfaces, and granulocyte-macrophage colony-stimulating factor (GM-CSF) was reported to be able to alter expression of TLRs and cytokine production in neutrophils. However, whether GM-CSF modulates the expression of TLR and cytokine production in mast cells is not clear.

**Results:**

Using flow cytometry and real time PCR techniques, we found that GM-CSF upregulated expression of TLR3 and TLR7 in P815 cells in a concentration dependent manner. GM-CSF also provoked approximately up to 2.4 and 2.3 fold increase in IL-13 and IL-6 release from P815 cells, respectively following 16 h incubation. GM-CSF induced IL-13 secretion, TLR3 and TLR7 expression appeared to be through activation of mitogen-activated protein kinase (MAPK) and phosphotidylinositol 3-kinase (PI3K)/Akt signaling pathways, whereas GM-CSF elicited IL-6 release seemed via Akt signaling pathway. At 10 ng/ml, GM-CSF significantly enhanced R-848-induced IL-6 release from P815 cells.

**Conclusion:**

The ability of GM-CSF in modulation of expression of TLR3 and TLR7 in P815 mast cells and in stimulation of IL-13 and IL-6 release from P815 mast cells in vitro suggests that GM-CSF might play an important role in enhancing the innate immune responses of mast cell to viral infection

## Background

GM-CSF is a cytokine which has been shown to actively participate in regulation of TLR expression and cytokine production in inflammatory cells. For example GM-CSF upregulates expression of TLR2 in human neutrophils and monocytes [1], elicits IL-8 release from neutrophils through TLR2 [2], and enhances expression of TLR4 [1] and TLR9 [3] in neutrophils. Administration of anti-GM-CSF antibody after LPS challenge effectively reduced neutrophil counts and endotoxin-induced TLR4 expression in the lungs of BALB/c mice [4], indicating that GM-CSF may contribute to a protective immunity against bacteria infection. As an active proinflammatory cytokine, GM-CSF can be generated by several cell sources including T and B lymphocytes, macrophages, keratinocytes, eosinophils, neutrophils, and mast cells [5]. The reports that human mast cells can produce substantial level of GM-CSF following bacterial PGN activation [6], and human cord blood-derived mast cells and human mast cell line (HMC-1) can release GM-CSF in response to IgE [7] or calcium ionophore A23187 [8], suggest that GM-CSF is likely to affect mast cell functions.

Mast cells have long been recognized as the primary effector cells of allergy [9]. However, recent insight into mast cells has revealed this cell type as key players in the regulation of innate [10] as well as adaptive immunity through TLRs [11,12]. It was found that Peptidoglycan (PGN) from Staphylococcus aureus stimulated bone marrow-derived mast cells in a TLR2-dependent manner to produce TNF-alpha, IL-4, IL-5, IL-6 and IL-13 [13-15], whereas LPS from Escherichia coli stimulated mast cells in a TLR4-dependent manner to produce TNF-alpha, IL-1beta, IL-6, and IL-13 [13,16-18]. Poly(I:C), R-848, and CpG oligodeoxynucleotide, which are TLR3, TLR7, and TLR9 activators was able to induce proinflammatory cytokines (TNF-alpha and IL-6) and chemokines (RANTES, MIP-1alpha, and MIP-2) release from murine fetal skin-derived cultured mast cells [19]. However, the mechanisms through which these TLR expressions on mast cells and cytokine release from mast cells were regulated remain poorly understood.

TLRs are a group of single membrane-spanning non-catalytic receptors that recognize structurally conserved pathogen-associated molecular patterns derived from microbes, and activate immune cell responses [20,21]. Among the 11 known TLRs, TLR3 has been shown to be present in human [22] and murine mast cells [23], which responds to viral double-stranded RNA and single-stranded RNA of selected species [22]. TLR7 has also been found in human [23] and murine mast cells [19], which can be recognized by synthetic imidazoquinoline as well as several single-stranded RNA sequences of viral origin [24]. Similarly, TLR9 have been located in human [25] or murine mast cells [19], which can be activated by DNA sequences that are rare in mammalian genomes but common in the genetic materials of bacteria, fungi, and DNA viruses [25]. Since TLRs are receptors for micro-organism pathogens, mast cells highly express them, GM-CSF can regulate TLR expression and cytokine production in inflammatory cells, we anticipate that GM-CSF ought to regulate TLR expression and cytokine production in mast cells, and through which participate in innate immunity against bacterial and viral invasion. We found that GM-CSF were able to upregulate expression of TLR3 and TLR7 on P815 mast cells and provoke IL-13 and IL-6 release from P815 mast cells in the present study.

## Results

### Expression of TLRs in P815 cells

**Figure 1:**
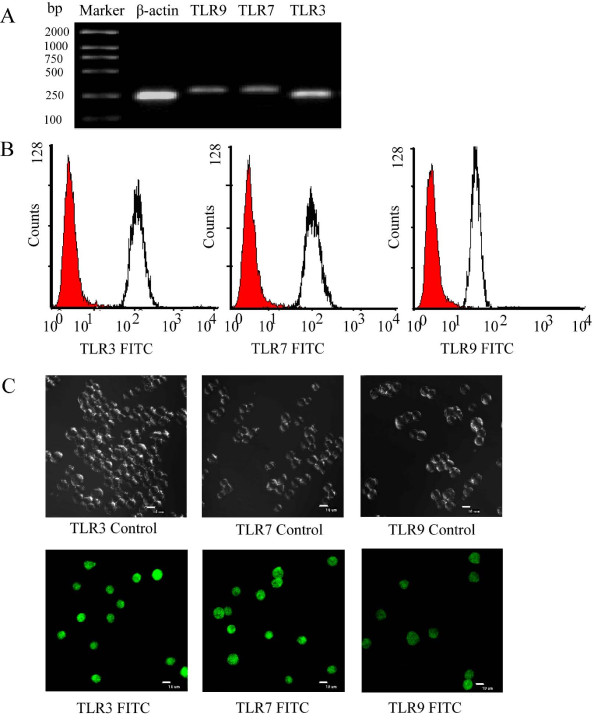
**Analysis of expression of TLRs on P815 cells**. (**A**) For RT-PCR analysis, the gene products were separated in a 1.5% agarose gel. Lane 1–5 represented DNA marker, β-actin (288 bp), TLR3 (240 bp), TLR7 (262 bp) and TLR9 (297 bp), respectively. (**B**) For flow cytometry analysis, cells were incubated with FITC-conjugated goat anti-mouse TLR9 monoclonal antibody or incubated with rabbit anti-mouse TLR3, TLR7 polyclonal antibodies at 37°C for 1 h. (**C**) For immunofluorescent microscopy, cells were treated with the above-mentioned procedures and analyzed with a laser scanning confocal microscope.

In order to ensure if P815 cells are the appropriate cells for the investigation of regulatory effect of GM-CSF on TLR expression, we first examine the expression of TLRs in these cells. With RT-PCR analysis, we showed that P815 cells express mRNAs of TLR3, TLR7 and TLR9 (Fig 1A). Using flow cytometry analysis (Fig 1B) and immunofluorescent cell staining (Fig 1C) techniques, we confirmed that P815 cells also express TLR3, TLR7 and TLR9 proteins.

### Modification of expression of TLRs in P815 cells by GM-CSF

**Figure 2:**
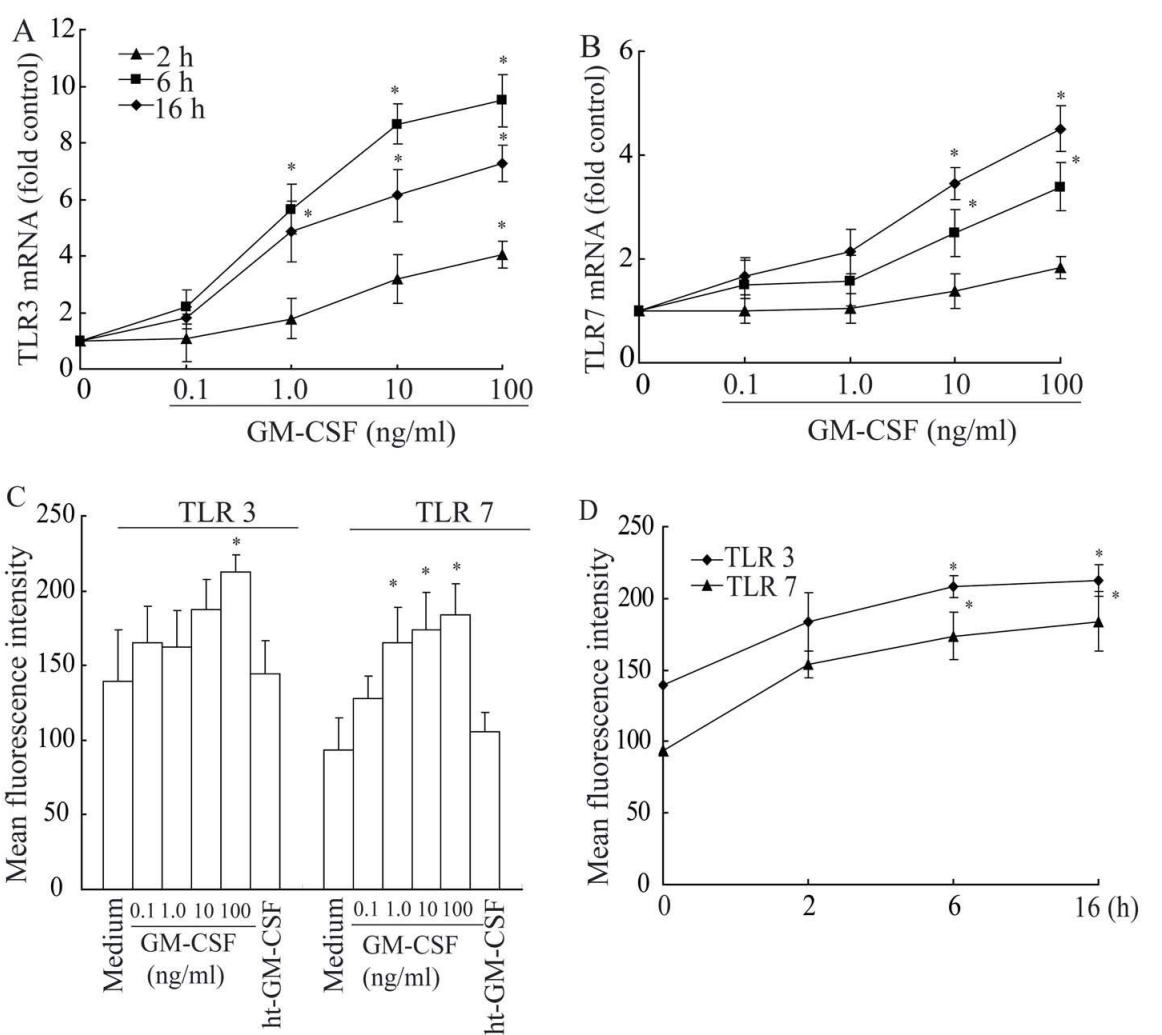
**Induction of upregulated expression of TLR3 (A), TLR7 (B) mRNAs and TLR3 (C), TLR7 (D) proteins in P815 cells by GM-CSF**. For real-time PCR analysis, cells were treated with various concentrations of GM-CSF at 37°C for 2, 6 and 16 h, respectively. The data were normalized to the housekeeping gene (β-actin gene) and were expressed as Mean ± SE fold over control for four separate experiments performed in duplicate. For flow cytometry analysis, the cells were incubated with various concentrations of GM-CSF for 16 h (**C**) or with 100 ng/ml of GM-CSF for 2, 6 and 16 h (**D**). ht-GM-CSF represented the heat-treated GM-CSF (100 ng/ml). Values shown are Mean ± SE for four separate experiments performed in duplicate. * *P *< 0.05 compared with the response to corresponding medium alone control.

In order to examine if GM-CSF induces altered expression of TLR3, TLR7 and TLR9 mRNA, quantitative real time RT-PCR was employed. The results showed that GM-CSF at 1.0 to 100 ng/ml up-regulated expression of TLR3 and TLR7 mRNAs in P815 cells in a concentration dependent manner. Up to approximately 9.5 fold increase in TLR3 mRNA expression was observed in P815 cells. GM-CSF induced up-regulation of TLR3 mRNA expression initiated at 2 h, peaked at 6 h, and declined at 16 h following incubation (Fig. 2A). GM-CSF provoked enhancement of TLR7 mRNA expression appeared a slow process, and a dose dependent curve was achieved only at 16 h following incubation. Up to approximately 4.5 fold increase in expression of TLR7 mRNA was observed in P815 cells (Fig. 2B). GM-CSF at the concentrations tested had little effect on TLR9 mRNA expression (data not shown).

We then used flow cytometry analysis technique to determine if GM-CSF stimulates increased expression of TLR3 and TLR7 proteins. The results showed that GM-CSF at 0.1 to 100 ng/ml provoked upregulation of expression of TLR3 and TLR7 in P815 cells in a dose dependent manner following 16 h incubation period. Approximately up to 52% and 96.3% upregulated expression of TLR3 and TLR7 was observed when cells were incubated with 100 ng/ml of GM-CSF for 16 h (Fig 2C). The heat treatment completely abolished the ability of GM-CSF in upregulation of TLR3 and TLR7 protein expression (Fig 2C). The time course study showed that significant up-regulation of expression of TLR3 and TLR7 by GM-CSF was first observed at 6 h, and lasted at least to 16 h following incubation (Fig 2D). In the parallel experiments, immunofluorescent analysis showed similar pattern of increased expression of TLR3 and TLR7 in P815 cells following 2, 6 and 16 h incubation periods (data not shown). With flow cytometry analysis and immunofluorescent staining techniques, it was shown that GM-CSF failed to alter TLR9 expression in P815 cells (data not shown).

### Induction of cytokine secretion from P815 cells by GM-CSF

**Figure 3:**
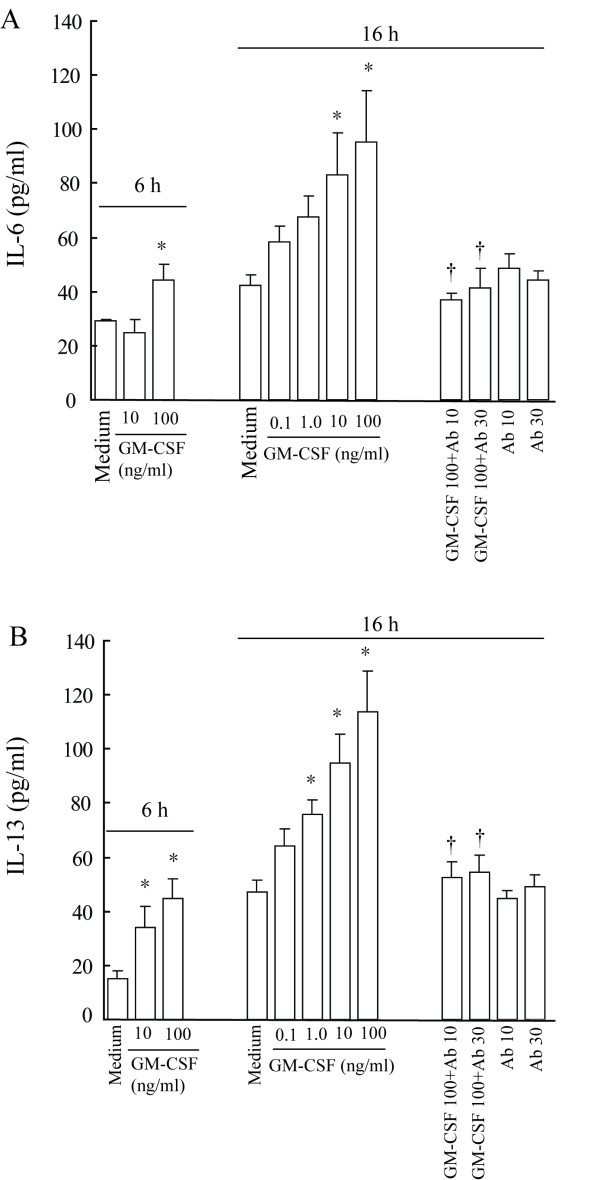
**Induction of IL-6 (A) and IL-13 (B) release from P815 cells by GM-CSF**. Cells were incubated with various concentrations of GM-CSF (ng/ml) or GM-CSF with its blocking antibody (Ab, μg/ml) at 37°C for 6 and 16 h. Values shown are Mean ± SE for four separate experiments performed in duplicate. * *P *< 0.05 compared with the response to corresponding medium alone control.

GM-CSF has been shown to elicit IL-8 release from neutrophils through TLR2. However, little is known of the ability of GM-CSF in induction of cytokine release from mast cells. In order to examine if GM-CSF can induce cytokine release from mast cells, P815 cells were challenged with GM-CSF, and levels of IL-6, IL-12 and IL-13 were measured. They represent proinflammatory cytokines (IL-6), Th1 cytokines (IL-12) and Th2 cytokines (IL-13), respectively. The results showed that GM-CSF at 0.1 to 100 ng/ml induced a concentration dependent release of IL-6 from P815 cells following 16 h incubation. Approximately up to 2.3 fold increase in IL-6 release was achieved when 100 ng/ml of GM-CSF was incubated with cells (Fig 3A). Similarly, GM-CSF provoked approximately up to 2.4 fold increase in IL-13 release from P815 cells following 16 h incubation (Fig 3B). Preincubation of anti-GM-CSF antibody with cells for 30 min significantly eliminated GM-CSF induced IL-6 and IL-13 secretion (Fig 3A, B). GM-CSF also elicited significant release of IL-6 (Fig 3A) and IL-13 (Fig 3B) from P815 cells at 6 h following incubation. GM-CSF at the concentrations examined had little effect on IL-12 secretion from P815 cells (data not shown).

### Effect of GM-CSF on poly (I:C) and R-848-induced IL-6 release from P815 cells

**Figure 4:**
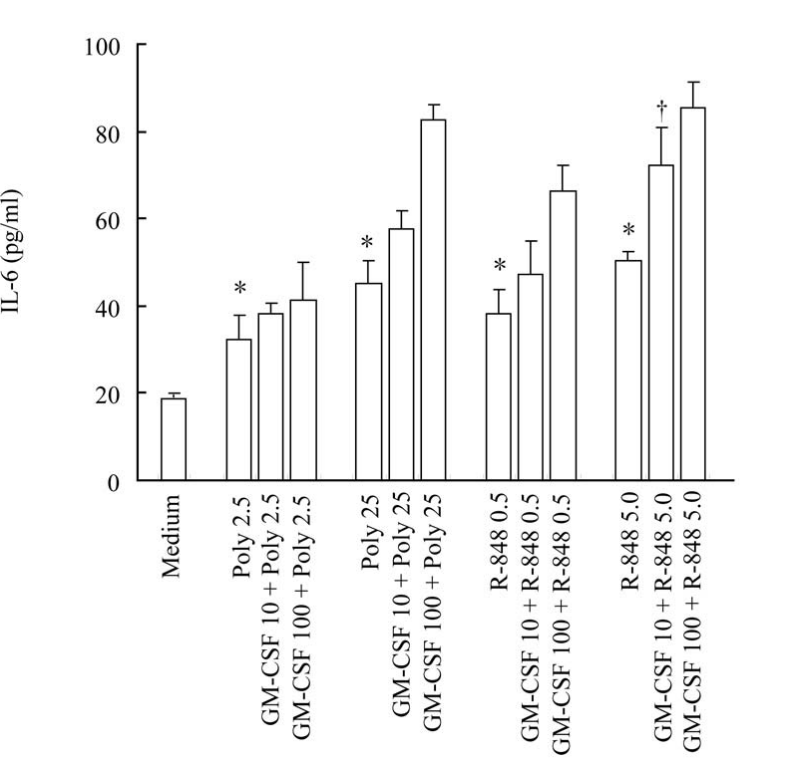
**Effect of GM-CSF on poly (I:C) and R-848 induced IL-6 secretion from P815 cells**. The cells were preincubated with GM-CSF for 1 h before adding poly (I:C) or R-848 for 6 h. Values shown are Mean ± SE for four separate experiments performed in duplicate. * *P *< 0.05 compared with the response to corresponding medium alone control. † *P *< 0.05 compared with the response to R-848 5.0 μg/ml alone. Poly = poly (I:C).

In order to determine if GM-CSF affect poly (I:C) and R-848 induced IL-6 release. P815 cells were preincubated with GM-CSF for 1 h before adding Poly (I:C) and R-848 for 6 h. The results showed that GM-CSF at 10 ng/ml significantly enhanced R-848-induced IL-6 release, and at 100 ng/ml had additive effect on poly (I:C) and R-848-induced IL-6 release from P815 cells (Fig 4).

### Effect of cell signaling inhibitors on GM-CSF-induced release of cytokines and upregulated expression of TLR3 and TLR7

Little information on GM-CSF signal pathways of mast cells is available, but the findings that GM-CSF modulates neutrophil response to bacterial DNA by activation of the mitogen-activated protein kinase (ERK)1/2 [26], and that IL-12 induced IL-4 release through activation of ERK and Akt signaling pathways in P815 mast cell line [27] may give us a clue to explore the possible signaling pathways in P815 cells. We therefore employed PD98059 a MAPK pathway inhibitor, U0126 an inhibitor of MEK and thus a MAPK pathway inhibitor, SB203580 a selective inhibitor of p38 MAPK, LY294002 a PI3K inhibitor and AG490 a Janus kinase (JAK)/STAT3 pathway inhibitor to investigate the potential GM-CSF signal pathways in P815 cells.

**Figure 5:**
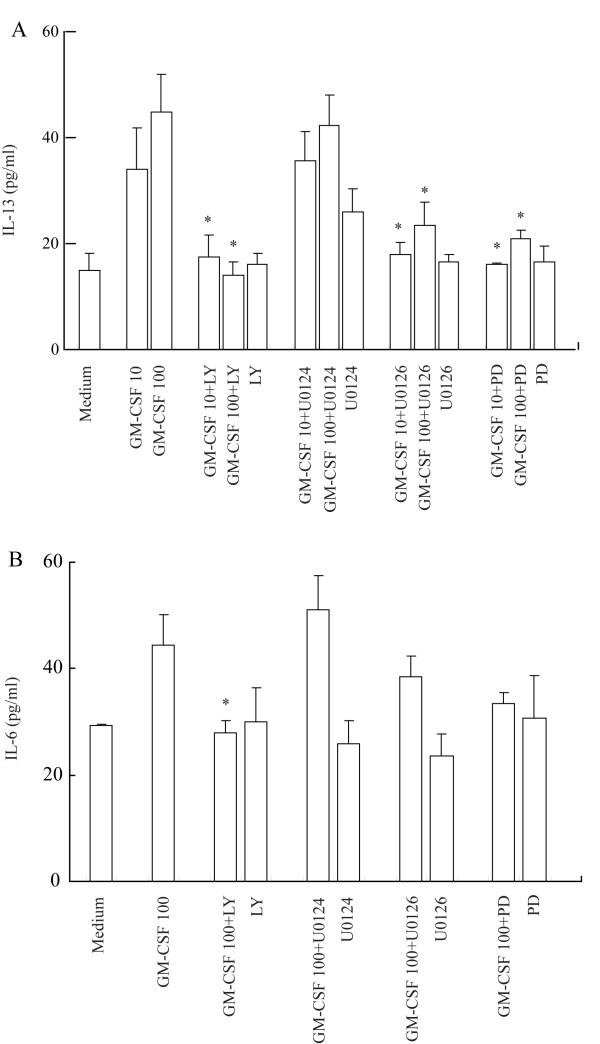
**Effect of PD98059 (PD), U0126, U0124 and LY294002 (LY) on GM-CSF induced IL-13 (A) and IL-6 (B) secretion from P815 cells**. Cells were preincubated with PD (50 μM), U0126 (5 μM), U0124 (5 μM) or LY (20 μM), respectively for 30 min before GM-CSF (10 and 100 ng/ml) being added for 6 h at 37°C. Values shown are Mean ± SE for four separate experiments performed in duplicate. * *P *< 0.05 compared with the response to corresponding uninhibited control.

**Figure 6:**
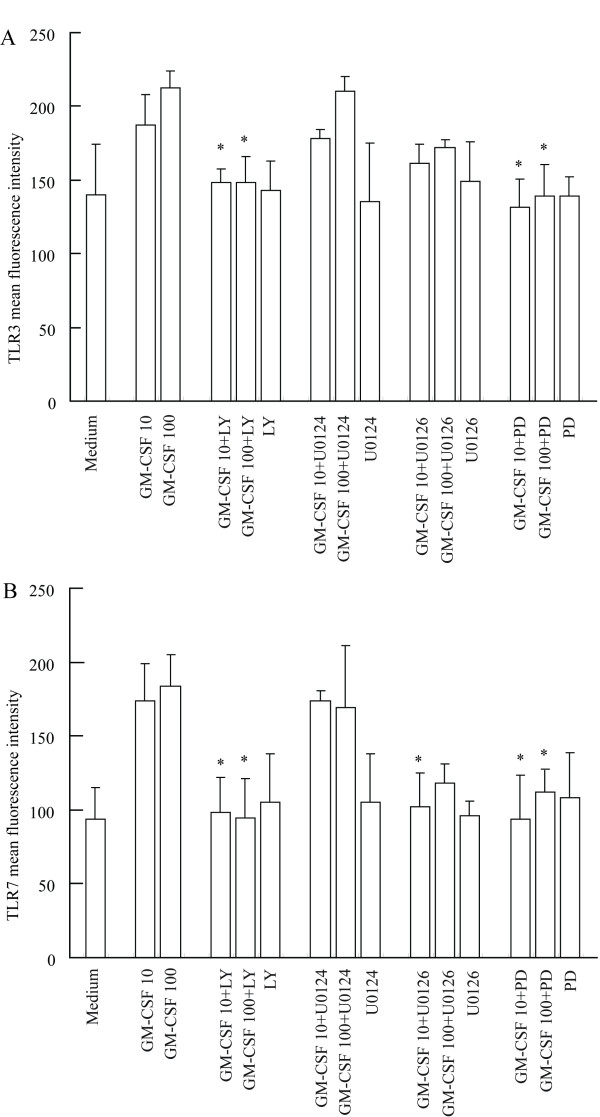
**Effect of PD98059 (PD), U0126, U0124 and LY294002 (LY) on GM-CSF upregulated TLR3 (A) and TLR7 (B) expression in P815 cells**. Cells were preincubated with PD (50 μM), U0126 (5 μM), U0124 (5 μM) or LY (20 μM), respectively for 30 min before GM-CSF (10 and 100 ng/ml) being added for 6 h at 37°C. Values shown are Mean ± SE for four separate experiments performed in duplicate. * *P *< 0.05 compared with the response to corresponding uninhibited control.

PD98059, U0126 and LY294002 almost completely abolished GM-CSF-induced IL-13 release from P815 cells when they were preincubated with the cells for 30 min (Fig 5A), indicating that GM-CSF induced IL-13 release is through activation of MAPK and PI3K/Akt signaling pathways. In contrast, SB203580, U0124 a structural analogue negative control of U0126 and AG490 had little influence on GM-CSF induced IL-13 release (data not shown). LY294002 also completely abolish GM-CSF induced IL-6 release (Fig 5B), indicating that GM-CSF induced IL-6 release is through activation of PI3K/Akt signaling pathway. All inhibitors tested did not significantly affect basal IL-6 and IL-13 release. PD98059 also completely abolished GM-CSF induced upregulation of TLR3 (Fig 6A) and TLR7 (Fig 6B) expression, and U0126 eliminated GM-CSF induced enhancement of TLR7 expression, indicating that the actions of GM-CSF are via activation of MAPK signaling pathway. Similarly, LY294002 completely abolish GM-CSF induced upregulation of TLR3 and TLR7 expression (Fig 6), indicating GM-CSF induced upregulation of TLR3 and TLR7 expression is through activation of PI3K/Akt signaling pathway. As expected, SB203580, U0124 and AG490 had little influence on GM-CSF induced upregulation of TLR3 and TLR7 expression (data not shown). All inhibitors tested did not significantly affect basal TLR3 and TLR7 expression in P815 cells.

### Inhibition of GM-CSF-induced phosphorylation of ERK and Akt by signaling inhibitors

PD98059 and U0126 inhibited approximately up to 50 and 46.7% of GM-CSF induced phosphorylation of ERK, and LY294002 diminished GM-CSF-induced phosphorylation of Akt by approximately 76.2%, respectively in P815 cells following 30 min preincubation period.

## Discussion

It was found for the first time that GM-CSF was able to upregulate expression TLR3 and TLR7 and to stimulate IL-13 and IL-6 from mast cells. Since MAPK pathway inhibitors and PI3K inhibitor inhibited GM-CSF induced expression of TLR3 and TLR7 as well as IL-13 and IL-6 secretion, it is most likely that GM-CSF induced IL-13 and IL-6 secretion is through TLR3 and TLR7 related mechanisms. GM-CSF could also prime the cells to respond to R-848 a TLR7 ligand stimulation. The previous findings that TLR3 [22], TLR7 [22] and TLR9 [25] are present in mast cells, and that GM-CSF can regulate expression of TLR2 and TLR4 in neutrophils [1-3] support our observation above.

As the main TLR related to the viral recognition, TLR3 have been shown to be able to respond to viral RNAs of selected species, and resulted in production of IFN-β and RANTES/CCL5 that are critical in regulating T-cell functions [28]. Moreover, TLR3-activated mast cells elicited strong chemotactic responses to CD8+ T cells in vitro and in vivo [29]. Like TLR3, TLR7 has also been demonstrated to recognize several single-stranded RNA sequences of viral origin. TLR7 activators, R-848 could activate murine mast cell to release proinflammatory cytokine (TNF-α and IL-6) and chemokines (RANTES, MIP-1α and MIP-2), and these factors, in turn, can contribute to antiviral effects [19]. Recent study found that mucosal activation of TLR7 by virus is one of the main mechanisms for the mast cell-dependent anorexia and hypothermia [24]. Since TLR3 and TLR7 play critical roles in innate immune recognition of virus, our results suggest that GM-CSF is likely to play a promising role as immunodulator for enhancing virus recognition by mast cells.

To confirm the potential pivotal role of GM-CSF in immunity, we measured the levels of IL-12, IL-13 and IL-6 in GM-CSF treated cell supernatant. Instead of induction of IL-12 production, GM-SCF elicited a significant IL-13 release from P815 cells. Since IL-13 is emerging as an important mediator in the development of Th2 cell responses, which can induce IgE secretion from activated human B cells [30] and acts more prominently as a molecular bridge linking allergic inflammatory cells to the non-immune cells [31], we believe that GM-SCF also play a role in acquired immune response such as allergic reactions. GM-SCF has been implicated in allergic inflammation in the previous studies. It was reported that a higher proportion of cells expresses GM-CSF mRNA in bronchoalveolar lavage (BAL) fluid from asthma patients compared with healthy controls [32]. When asthma patients were challenged with allergen, GM-CSF levels increased in the BAL fluid, and the levels correlated with the number and percentage of BAL eosinophils [33]. Administration of a GM-CSF-neutralizing mAb attenuated allergic airway inflammation in a murine model of asthma, significantly reducing airway hyperresponsiveness, airway eosinophilia, and pulmonary inflammation [34]. IL-6 has long been recognized as a potent proinflammatory cytokine, which can be released from activated mast cells [35] and contributes to local inflammation and vessel expansion in airway walls of asthmatics [36]. Induction of IL-6 from P815 cells by GM-SCF proves further that GM-CSF participates in the pathogenesis of inflammation.

GM-CSF induced IL-13 secretion appeared to be through activation of MAPK and PI3K/Akt signaling pathways, whereas GM-CSF elicited IL-6 release seemed via PI3K/Akt signaling pathway as MAPK pathway inhibitors or PI3K inhibitor inhibited GM-CSF induced IL-13 or IL-6 secretion. While little information on GM-CSF signal pathways of mast cells is available, a study which showed that GM-CSF modulates the CpG-independent, MyD88-dependent neutrophil response to bacterial DNA, by increasing the activation of the ERK1/2 [26] may help to understand our above observations. The work demonstrated that C3a stimulated substantial MCP-1 and RANTES/CCL5 production, and ERK and Akt phosphorylation in human LAD 2 mast cells [37]; and our former study that IL-12 induced IL-4 release through activation of ERK and Akt signaling pathways in P815 mast cell line [27] may support our current findings.

## Conclusion

The ability of GM-CSF in modulation of expression of TLR3 and TLR7 in P815 mast cells and in stimulation of IL-13 and IL-6 release from P815 mast cells in vitro suggests that GM-CSF might play an important role in enhancing the innate immune responses of mast cell to viral infection

## Methods

### Reagents and cells

Paraformaldehyde, bovine serum albumin (BSA, fraction V) and 4-(4-fluorophenyl)-2-(4-methylsulfinylphenyl)-5-(4-pyridyl)-1H-imidazole (SB203580) were from Sigma Inc. (St Louis, MO, USA). Recombinant mouse GM-CSF, anti-mouse GM-CSF monoclonal antibody (mAb) was from R&D Systems (Minneapolis, MN, USA). Tissue culture reagents including Dulbecco's modified Eagle's medium (DMEM), HEPES and fetal bovine serum (FBS) were obtained from GibcoBRL (Carlsbad, CA, USA). Cellular activation of signaling ELISA CASE kits for Akt, ERK, p38 MAPK and signal transducer and activators of transcription (STAT)3 were from SuperArray Bioscience Corporation (Frederick, MD, USA). Mouse IL-6, IL-12 and IL-13 ELISA kits were from Pierce Biotechnology Inc. (Rockford, IL, USA). 2-(2-Diamino)-3-methoxyphenyl-4H-1-benzopyran-4-one (PD98059), 1,4-diamino-2,3-dicyano-1,4-bis(2-aminophynyltio)butadiene (U0126), tyrphostin (AG490), 1,4-diamino-2,3-dicyano-1,4-bis(methylthio) butadiene (U0124) and 2-(4-morpholinyl)-8-phenyl-4H-1-benzopyran-4-one (LY294002) were from Cell Signaling Technology (Beverly, MA, USA). TRIzol Reagent and SYBR Green I Stain were from Invitrogen (Carlsbad, CA, USA). ExScript RT reagent kit and SYBR Premix Ex Taq (perfect real time) was from TaKaRa Biotechnology Co. Ltd (DaLian, China). FITC-conjugated rat anti-mouse TLR9 mAb, FITC-conjugated rat isotype control, rabbit anti-mouse TLR3 and TLR7 mAbs were from eBioScience (Los Angeles, CA, USA). FITC-conjugated goat anti-rabbit polyclonal antibody was from BD Pharmingen (San Jose, CA, USA). Poly (I:C) and R-848 were from Invivogen (San Diego, CA, USA). The mouse mastocytoma cell line (P815) was obtained from the American Type Culture Collection (ATCC, Manassas, VA, USA). Most of other reagents such as salt and buffer components were analytical grade and obtained from Sigma.

### P815 cell culture and challenge

P815 cells were cultured with ATCC complete growth medium including DMEM with 4 mM L-glutamine, 1.5 mg/ml sodium bicarbonate, 4.5 mg/ml glucose, 10% FBS, 100 U/ml penicillin and 100 mg/ml streptomycin in 75-cm^2 ^tissue culture flasks (Falcon) at 37°C in a 5% (v/v) CO_2_, water-saturated atmosphere. P815 cells at a density of 1 × 10^6 ^cells/ml were incubated with the serum-free basal medium for 6 h and washed twice before challenge. For challenge experiments, cells were exposed to various concentrations of GM-CSF (0.1–100 ng/ml) with or without its blocking antibody (10, 30 μg/ml). Heat-treated GM-SCF (ht-GM-CSF) was prepared by incubation of GM-SCF at 100°C for 10 min, and was used as irrelevant protein control for challenge experiment. At 2, 6 or 16 h following incubation, the culture plates were centrifuged at 450 g for 10 min at 25°C. After the supernatant (5 ml) being collected and stored at -80°C, the cell pellet containing approximately 5 × 10^6 ^cells were resuspended for immunofluorescence and real-time PCR analysis.

For certain experiments, cells were preincubated with 10 ng/ml and 100 ng/ml of GM-CSF for 1 h before adding 2.5 and 25 μg/ml of poly (I:C) or 0.5 and 5.0 μg/ml R-848. At 6 h following incubation, the culture plates were centrifuged at 450 g for 10 min at 25°C. The culture supernatants were collected and stored at -80°C for further use.

For cell signalling experiments, cultured cells at a density of 1.5 × 10^6 ^cells/ml were washed twice with the serum-free basal medium and then treated with the inhibitors of cell signalling pathways including PD98059 (50 μM), U0126 (5 μM), U0124 (5 μM), SB203580 (20 μM), LY294002 (20 μM) and AG490 (40 μM) for 30 min before being challenged with GM-CSF (10 and 100 ng/ml) for 15 min, 2 or 6 h. Following incubation, 400 μl of cell suspension from each well was removed for signaling ELISA analysis, and the remaining 700 μl of cell suspension was centrifuged at 450 g for 10 min at 25°C. The culture supernatants were then collected and stored at -80°C, and cells (~1 × 10^6^) were resuspended for immunofluorescence analysis.

### Examination of expression of TLR mRNAs

The expression of TLR mRNAs in P815 cells was determined with RT-PCR. Total RNA was isolated by using a TRIzol reagent kit according to the manufacturer's instruction. Briefly, cells were collected by centrifugation and lysed directly by adding TRIzol reagent (1 ml per 1 × 10^6 ^cells). After being treated with chloroform, RNA was precipitated by adding 0.5 ml of isopropyl alcohol and then resuspended with 1 ml of 75% (v/v) ethanol. Total RNA was quantified by measuring absorbance ratios at 260/280 nm. The cDNA was prepared by reverse transcriptase using a commercial RNA-PCR kit according to the manufacturer's instruction. For each reaction, 1 μg of total RNA was reversely transcribed using oligo-d (T). The cDNA was amplified using forward and reverse specific primers for amplifying mouse TLRs. β-actin was used as an internal control. Primers were designed according to the genbank sequences for mouse TLRs and summarized in Table 1. The conditions for amplification were as follows: 95°C for 5 min, 30 cycles of denaturation at 95°C for 30 s, annealing temperatures as shown in Table 1 for 30 s, and extension at 72°C for 30 s. PCR products were electrophoresed on 1.5% agarose gels that were stained with SYBR Green I Nucleic Acid Gel Stain and photographed under ultraviolet (UV) light.

**Table 1:** Primers for RT-PCR and real-time RT-PCR

Target gene	Forward sequence (5'-3')	Reverse sequence (5'-3')	AS (bp)	AT (°C)	GA
TLR3	GGTGGTCCCGTTAATTTCCT	CCCGAAAACATCCTTCTCAA	240	56	NM_126166
TLR7	TGGAAATTTTGGACCTCAGC	TTGCAAAGAAAGCGATTGTG	262	52	NM_133211
TLR9	TGCAGGAGCTGAACATGAAC	TAGAAGCAGGGGTGCTCAGT	297	58	NM_031178
β-actin	GCTACAGCTTCACCACCACAG	GGTCTTTACGGATGTCAACGTC	288	60	NM_007393

AS = amplicon size; AT = annealing temperature; GA = genbank accession

### Quantitative real-time PCR

Quantitative expression of TLR mRNAs in P815 cells was determined by real-time PCR following the manufacture's protocol. Briefly, after synthesizing cDNA from 1 μg of total RNA by using ExScriptTM RT reagent kit, real-time PCR was performed by using SYBR^® ^Premix Ex Taq TM on the ABI Prism 7000 Sequence Detection System (Perkin Elmer Applied Systems, Foster City, CA, USA). Each reaction contains 12.5 μl of 2 × SYBR green Master Mix, 1 μl of 10 μM of primers, 1 μl of the cDNA, to a total volume of 25 μl. The thermal cycling conditions included an initial denaturation step at 50°C for 2 min, 95°C for 10 min; 40 cycles at 95°C for 15 s, annealing temperatures as shown in Table 1 for 30 s and extension at 72°C for 30 s. Consequently, at the end of the PCR cycles, specificities of the amplification products were controlled by dissociation curve analysis. mRNA expression in each sample was finally determined after correction with β-actin expression. The gene specific threshold cycle (Ct) for each sample (ΔCt) was corrected by subtracting the Ct for the housekeeping gene β-actin. Untreated controls were chosen as the reference samples, and the ΔCt for all experimental samples were subtracted by the ΔCt for the control samples (ΔΔCt). The magnitude change of test gene mRNA was expressed as 2^-ΔΔCt^. Each measurement of a sample was conducted in duplicate.

### Flow cytometry analysis

P815 cells were pelleted by centrifugation at 450 g for 10 min, and then fixed in 2% paraformaldehyde for 30 min. After washing, the cells were resuspended in phosphate-buffered saline (PBS). For TLR9 staining, cells were incubated with FITC-conjugated rat anti-mouse TLR9 mAb or FITC-conjugated rat isotype control (all at a final concentration 4 μg/ml) at 37°C for 1 h. For TLR3 and TLR7 staining, cells were incubated with rabbit anti-mouse TLR3 and TLR7 polyclonal antibodies or normal rabbit IgG, respectively (all at a final concentration 2 μg/ml) at 37°C for 1 h. After two washes with 1% BSA/PBS, cells were incubated with 1 μg/ml of FITC-conjugated goat anti-rabbit polyclonal antibody at 37°C for 1 h. Cells were finally resuspended in PBS and analyzed on a FACSCalibur flow cytometer with CellQuest software (BD Biosciences).

### Immunofluorescent cell staining

After being fixed in 2% paraformaldehyde for 30 min, P815 cells were incubated with 3% normal serum blocking solution for 10 min. The same staining procedures were used as for the flow cytometry analysis. For TLR9 staining, cells were incubated with FITC-conjugated rat monoclonal antibody against mouse TLR9, or FITC-conjugated rat isotype control (all at a final concentration 4 μg/ml) at 37°C for 1 h. For TLR-3 and TLR7 staining, cells were incubated with rabbit anti-mouse TLR-3 and TLR7 polyclonal antibodies or normal rabbit IgG, respectively (all at a final concentration 2 μg/ml) at 37°C for 1 h. After two washes with 1%BSA/PBS, cells were incubated with 1 μg/ml of FITC-conjugated goat anti-rabbit polyclonal antibody at 37°C for 1 h. Images were obtained on a Zeiss 5 LIVE confocal laser scanning microscope (Zeiss, German).

### Determination of optimal concentration of inhibitors for signal transduction

To determine the optimal concentrations of the inhibitors of signal-transduction pathways in P815 cells, 10, 20, 25, 50 and 100 μM of PD98059, 2, 2.5, 5 and 10 μM of U0126, 10, 20, 40 and 80 μM of SB203580, 5, 10, 20 and 40 μM of LY294002 and 10, 20, 40, 80 and 160 μM of AG490 were preincubated with P815 cells for 30 min before adding stimulus. Since 50 μM of PD98059, 5 μM of U0126, 20 μM of SB203580, 20 μM of LY294002 and 40 μM of AG490 almost completely abolished, GM-CSF-induced phosphorylation of ERK1/2, p38 MAPK, Akt and STAT3, respectively (data not shown), and induced minimum cell death, they were chosen as the optimal concentration throughout the study.

### Determination of levels of cytokines

IL-6, IL-12 and IL-13 levels were measured by using ELISA kits, according to the manufacturer's instruction.

### Analysis of signal-transduction pathways

The phosphorylation of Akt, ERK, p38 and STAT3 was analyzed by using Cellular Activation of Signaling ELISA (CASE) kits as described previously [27].

### Statistics

Data are expressed as mean ± SEM for the indicated number of independently performed duplicated experiments. Statistical significance between means was analyzed by one-way analysis of variance or the Student's *t *test utilizing the SPSS 13.0 version. *P *< 0.05 was taken as statistically significant.

## Abbreviations

(TLRs): Toll-like receptors; (GM-CSF): granulocyte-macrophage colonystimulating factor; (MAPK): mitogenactivated protein kinase; (ERK): extracellular signal-regulated kinase; (STAT): signal transducer and activators of transcription; (RT-PCR): reverse transcription-polymerase chain reaction.

## Authors' contributions

HY carried out the most of experimental work and drafted the manuscript and supplied all analyzed data. JW participated in Real-time PCR experiments. HZ and LL participated in the cellular experiments and ELISA assay. WZ participated in Real-time PCR experiments. SH designed the experiments, raised the funds, coordinated the study, sorted out technical problems in the study and corrected the manuscript. All authors read and approved the final manuscript.
